# Supplementary material for: Are carbon emissions trading and green financial instruments synergistic? -Comprehensive quantitative research based on content analysis
Source: PLoS One. 2024 Mar 7;19(3):e0298601. doi: 10.1371/journal.pone.0298601 (PMC10919601; doi:10.1371/journal.pone.0298601)
Supplement: S1 Data — (ZIP) [file pone.0298601.s001.zip › Data/地级市Z_F工作报告.zip!/╜¡╦╒/╨∞╓▌╩╨/└┤╫╘╣1⁄2╓┌║┼í╛┬φ┐╦╩2╛▌═°í┐_2019.docx]

各位代表：

现在，我代表市人民政府向大会作工作报告，请予审议，并请市政协各位委员和其他列席人员提出意见。

一、2018年工作回顾

刚刚过去的2018年，是徐州发展史上极不寻常的一年，是外部环境复杂多变、经济下行压力加大、发展遇到诸多矛盾困难的一年，也是我们进一步匡正发展理念、夯实发展基础、加快转型升级的一年。一年来，在市委的坚强领导下，我们坚持以习近平新时代中国特色社会主义思想为指导，认真贯彻党的十九大精神，坚持稳中求进工作总基调，切实贯彻新发展理念，克难奋进、开拓创新，基本完成了十六届人大二次会议确定的目标任务。我们以贯彻落实习总书记视察徐州重要指示为主线，逐项明确时间表、路线图、任务书和牵引性工作抓手，系统性专题化推进指示精神落地生根，在发展实体经济、抓好创新驱动、推动乡村振兴、建设生态文明等方面拿出了一系列过硬措施，交出了阶段性合格答卷。我们以推动高质量发展为主旋律，全面落实省委“六个高质量”要求，更加注重调结构、强基础、补短板、增后劲、惠民生，经济社会发展总体平稳、稳中有进、稳中向好，全市实现地区生产总值（预计，下同）7000亿元左右，产业结构调整明显加快，经济发展质效明显提升、后劲明显增强，产业项目实现量质双升，污染防治取得显著成效，为下一步发展打下了坚实基础。我们以建设淮海经济区中心城市为主抓手，聚力建设“四个中心”，持续增强产业支撑力、城市承载力、发展竞争力和辐射带动力，徐州荣膺联合国人居奖，成功举办“世界城市日”中国主场活动，成为国务院表彰的老工业基地调整改造真抓实干成效明显城市，获国家节能减排示范市考核优秀等次，徐州资源枯竭城市转型发展经验全国推广，跻身国家创新型城市、国家知识产权示范市，通过全国文明城市和国家森林城市复检，淮海经济区协同发展机制初步建立，中心城市地位进一步巩固！

（一）产业强市建设迈出坚实步伐。牢固树立“工业立市、产业强市”的鲜明导向，高质量发展根基进一步夯实。产业结构调整步伐加快。全面落实装备与智能制造、新能源、集成电路与ICT、生物医药与大健康四大新兴主导产业实施方案，新兴产业加快发展，其中高端装备制造业产值增长32.9%。下决心实施钢铁、焦化、水泥、热电四大行业布局优化和转型升级，工业技改投资增长8.5%，占工业投资的51%；高耗能行业产值占比降低1.7个百分点，投资下降17.3%。扎实推进国家服务业综合改革试点，社会消费品零售总额增长8%，服务外包合同额、执行额分别增长194%和222%。财政资金较好发挥了“四两拨千斤”作用，市产业引导基金累计出资24.1亿元，设立基金29支，完成投资99.8亿元，撬动社会资本超过350亿元。产业项目建设扎实推进。制定“六项制度”和“765”计划，每两个月召开一次观摩点评会，在全市形成了抓招商、上项目的浓厚氛围。新签约注册项目720个，总投资3520亿元；开工、投产项目分别完成620个和460个，项目数量、质量、规模明显提升。固定资产投资完成3380亿元，投资结构进一步优化。经济运行质效稳步提升。工业开票销售完成4369.5亿元、增长6%，服务业应税销售完成7169亿元、增长26%。新增“四上”企业897家，其中规模以上工业企业276家。实现一般公共预算收入526.2亿元、增长4.9%，税占比79.2%、同比提高6.4个百分点。金融机构存贷款余额分别增长11.1%和17.7%，增速均居全省前列。民营经济较快发展，新登记各类市场主体18.2万户、增长9.8%。

（二）改革开放创新深入推进。坚持改革推动、开放带动和创新驱动，高质量发展动力显著增强。重点领域改革有序实施。供给侧结构性改革深入推进，完成省定去产能任务，房地产市场平稳健康，降低实体经济成本90亿元，实施补短板工程126项。“放管服”改革深入实施，“不见面”审批、“3550”、“证照分离”改革走在全省前列。市县机构改革积极酝酿，生产经营类事业单位、市区农商行等改革稳步推进。经营性国有资产实现统一监管，徐工混改、五大国资集团组建有序推进。农村各项改革全面深化，形成“1+N”的配套改革计划。全国河湖管护体制机制创新试点通过国家验收，全国水流产权确权试点通过中期评估。获评全国居家和社区服务改革试点优秀城市、国家首批流通领域现代供应链体系建设重点城市、全省国土资源节约集约利用模范市。出台全国首部地市级质量促进法规。双向开放空间加速拓展。徐州综保区实现封关运营，新沂保税物流中心通过联合验收，中欧班列突破100列，电子口岸、跨境电商等平台平稳运营。成功举办北京高质量发展恳谈会、“二十一洽”、智慧物流国际发展大会等重大招商活动，签约5亿元以上项目127个、3000万美元以上项目59个。徐州经济技术开发区综合实力大幅跃升，高新区实现进位，云龙、沛北经济开发区获批省级开发区，全市省级以上开发园区达到16家。实际使用外资18.8亿美元、增长13.3%；实现进出口总额785亿元、增长48.9%，其中出口654.3亿元、增长52.8%，增幅全省领先。精心组织首届进博会参会工作。创新引擎作用日益凸显。“一城一谷一区一院”四大创新平台加快建设，上海大院大所对接合作恳谈会、“赢在徐州”创新创业大赛成功举办，各类创新要素加速集聚。新增高新技术企业286家，是上年的2.09倍。新认定省级企业研发机构29家，新增省级科技企业加速器5家、跨境孵化器4家，新认定省级以上众创空间35家、科技企业孵化器15家。新引进诺贝尔奖获得者、两院院士等顶尖人才10人，千人计划、万人计划等领军人才46人，新入选省双创团队3个、双创人才28名、双创博士36人。邳州高新区、锡沂高新区正式获批省级高新区。徐工集团再获“中国工业大奖”。

（三）城乡融合发展格局加速构建。统筹推进中心城市建设、新型城镇体系构建、乡村振兴战略和区域协同发展，城乡一体化进程明显加快。中心城市功能加快提升。170项城建重点工程有序实施，淮海经济区金融服务中心开工建设，启动城市东南片区、会展中心片区规划编制，城市发展布局进一步优化。徐连高铁、徐宿淮盐高铁建设稳步推进，高铁动车所投入运营，徐宿连运河和徐洪河航道纳入省干线航道网规划，观音机场二期工程投入使用，航空客运量达252万人次、增长31.4%。轨道交通1、2、3号线建设进展顺利，迎宾大道高架主线贯通，立体交通体系加速构建。公共卫生中心等项目有序实施，五山公园一期等一批生态修复工程建成投用，公共服务设施更加完善。实施棚户区改造2346万㎡、拆迁扫尾项目进展顺利，完成老旧小区综合整治75个。国家全域旅游示范区创建全面启动，全年来徐游客量增长17.7%。市容环境综合整治深入推进，城市形象更加靓丽。乡村振兴战略开局良好。“1261”工作架构初步形成。粮食生产保持稳定，高效设施农业面积全省第一，农业科技服务体系加快完善。19个产业集群完成投资96亿元，新获批国家级龙头企业2家。新增上海蔬菜外延基地11家，总量达20家。乡村公共空间治理、农村人居环境整治、新型农民集中居住区建设、美丽宜居乡村创建等重点工作全面推开。6个村入选省特色田园乡村建设试点，1个村获评中国最美休闲乡村。新建改建农村公路729公里、桥梁315座，完成村内道路硬化1183公里，实施农村改厕4.76万户，城乡供水一体化工程进展顺利。区域协同发展机制初步形成。首届淮海经济区协同发展座谈会成功召开，在构建区域协同发展机制和交通互联互通、产业协同发展、环境保护联防联控、社会事业融合发展等方面达成战略合作协议，区域协同发展共识进一步凝聚、进程全面加快。5个中等城市功能提升和产城融合加快推进，9个试点中心镇创建有序实施，3个镇获评省乡村振兴优秀乡镇。

（四）三大攻坚战取得阶段性成效。动真碰硬推进污染治理、脱贫攻坚、风险防范，高质量发展难题加快破解。污染防治攻坚强力实施。开展大气污染防治攻坚行动，实施“六项治理”，共完成“散乱污”企业整治5257家，取缔217处非法石料加工点，扬尘污染治理和秸秆禁烧全面加强。市区PM2.5浓度61.6μg/m³、同比下降6.8%，优良天数比例56.2%、同比提升8个百分点，二氧化硫、氮氧化物减排量超额完成省定任务，空气质量改善幅度全省最大。“河长制”“断面长制”实现全覆盖，治理规模养殖场1743家，完成23条规划区黑臭水体整治、24条建成区黑臭水体景观提升，国考、省考断面水质实现全面达标。土壤风险评估和部分地块修复治理有序开展。精准脱贫攻坚进展良好。年内实现脱贫20.38万人，完成省定任务的146.6%；全市已有60万低收入人口实现脱贫，脱贫率88.5%。82个经济薄弱村集体年收入达到18万元以上，累计有218个经济薄弱村达标、完成任务的81%。全市256个经济薄弱村综合服务中心实现达标建设。通过市县财政补助完成4210户农村危房改造。丰县湖西片区群众生产生活条件明显改善。重大风险防范化解有力推进。政府债务底数基本摸清，隐性债务化解方案出台实施，政府债务总体可控。重点抓好8家农商行不良贷款清降，完成清降30亿元。防范和处置非法集资、互联网金融风险专项整治扎实开展，金融市场保持稳定。

（五）保障和改善民生力度不断加大。强化民生优先的工作导向，社会事业“十二大体系”加快构建，年度75件民生实事基本完成。城乡居民收入平稳增长。城乡居民人均可支配收入分别达到33600元、18200元，同比分别增长8.5%和9%。新增城镇就业12.99万人、农村劳动力转移5.6万人。开展创业培训1.79万人次，发放创业担保贷款2.18亿元。城镇登记失业率1.78%。社会保障水平稳步提高。“五险”扩面新增9.72万人，办理职工互助保险17.55万人。企业离退休人员养老金实现“14连调”。基本照护险实现提标扩面。城乡居民医保并轨运行。城乡低保标准分别提高到每人每月586元和550元。发放就业困难人员社保补贴1.53亿元、残疾人“两项补贴”2.93亿元。社会治理能力持续增强。扫黑除恶扎实开展，侦办40多个黑社会性质组织和恶势力犯罪集团。刑事案件、“八类案件”、“两抢”发案分别下降2.3%、9.53%和31.3%。安全事故起数、死亡人数分别降低10.39%和12.26%，重大特大事故实现“双杜绝”。进京、去省上访人次分别下降48.1%和7.1%。各项社会事业协调发展。新改扩建幼儿园216所、中小学133所，各学段教育教学质量明显提升，高考本科上线率提高5.4个百分点。新增三甲医院3家、院士工作站7家，改扩建村卫生室264所。第五届文博会、首届淮海书展成功举办，大运河文化带建设积极推进，64个镇（街道）文化站达标建成。徐州国际马拉松等品牌赛事影响力进一步提升，我市体育健儿在多项国际国内大赛上取得良好成绩。完成178家城市社区基本公共服务标准化建设。建成老年人日间照料中心57个、居家养老服务中心100个。市区59个农贸市场计量器具实现“六统一”。人防、国防动员、双拥、民族宗教、档案、外事、侨务、台湾事务、审计、统计、物价、食品药品监管、机关事务管理、住房公积金管理、气象、青少年、妇女儿童、广播电视等各项事业取得新进步。

（六）政府自身建设得到加强。积极解放思想、健全制度、提升效能，政府职能进一步转变。解放思想大讨论深入开展。系统化开展思想武装、定位提升、对标找差等“六大行动”，在全市持续掀起活动热潮，努力摆脱“地级市思维”、破除传统的“苏北意识”、卸下老工业基地“包袱”，为推进高质量发展凝聚了广泛共识。营商环境建设全面展开。围绕打造“六大环境”，出台“1+4”配套文件，实施“八大专项行动”和“五查五整治”，解决突出问题2000多个。出台支持民营经济健康发展意见，组建总规模20亿元的融资担保公司，开展“企业服务月”活动，有力提振了民营企业发展信心。政府效率效能不断提升。规范市政府常务会议、月度工作例会制度，健全重点难点事项协调机制，强化政务督查考核，政府运转更加高效，法治政府建设获评省优秀等次。建成数字化审图平台，实现投资项目建设一窗受理、分类审批、全程代办。取消各类证明事项396项，办理各类审批服务、便民服务102.8万件，12345政府服务热线受理群众诉求45万余件、一次办结率76.1%。办理市人大代表议案建议302件、市政协提案587件，办结率100%。党风廉政建设持续深化。严格执行中央八项规定精神及省、市委贯彻落实具体办法，严格落实“一岗双责”，切实防止“四风”反弹，专项治理发生在群众身边的不正之风和腐败问题，一批违法违纪人员受到严厉惩处，容错纠错机制进一步健全，全市上下干事创业的氛围更加浓厚！

各位代表！过去一年，面对各种困难挑战，我们迎难而上、主动作为，实现了徐州高质量发展良好开局！在此，我代表市人民政府，向全市人民，向各位人大代表、政协委员，向各民主党派、工商联、无党派人士，向各人民团体、各界人士，向驻徐人民解放军、武警官兵和人民警察，向关心和支持徐州建设的香港特别行政区同胞、澳门特别行政区同胞、台湾同胞、海外侨胞和国际友人，表示衷心的感谢和崇高的敬意！

我们也清醒地看到，徐州经济社会发展还存在诸多困难和问题：产业发展不充分，产业层次不高，结构调整阵痛正在显现，转型升级任重道远；经济下行压力加大，部分主要指标增速较年初预期有一定差距，一些企业生产经营困难，新动能培育还需加力提速；中心城市集聚辐射能力还不强，城乡二元结构明显；打赢污染防治攻坚战任务仍然十分艰巨，生态环境尚未根本好转；公共服务供给与群众日益增长的需求还不匹配；一些政府工作人员发展紧迫感不强、办事效率不高、主动担当作为意识有待提升，一些领域不正之风和腐败问题不容忽视，等等。对这些问题我们将高度重视，采取有力措施，切实加以解决。

二、2019年主要目标任务和重点工作

今年是新中国成立70周年，是决胜高水平全面建成小康社会的关键之年。随着“一带一路”建设、长三角一体化战略、淮河生态经济带发展规划、省“1+3”功能区战略深入实施，各种有利条件持续累积，特别是近年来我们狠抓招商引资、项目建设和营商环境打造，一批项目将陆续建设、投产，为徐州推动高质量发展提供强劲动力。同时，宏观经济形势错综复杂、不确定不稳定因素增加，加之徐州正处在转型升级、爬坡过坎的关键阶段，制约高质量发展的矛盾问题仍然突出。我们既要看到有利条件和积极因素，又要充分估计面临的困难和挑战，主动适应新时代要求、高质量发展要求，做到变中求稳有“进”的信心、优化结构有“稳”的定力、走在前列有“新”的作为、解放思想有“深”的拓展，努力在真抓实干中赢得发展主动权。

今年政府工作的指导思想是：坚持以习近平新时代中国特色社会主义思想为指导，全面贯彻党的十九大和十九届二中、三中全会精神，以贯彻落实习近平总书记视察徐州重要指示为主线、以推动高质量发展为主旋律、以建设淮海经济区中心城市为主抓手，统筹推进“五位一体”总体布局，协调推进“四个全面”战略布局，坚持稳中求进工作总基调，坚持新发展理念，坚持推动高质量发展，坚持深入推进供给侧结构性改革，坚持深化市场化改革、扩大高水平开放，加快构建现代化经济体系，继续打好三大攻坚战，认真做好稳就业、稳金融、稳外贸、稳外资、稳投资、稳预期工作，推动淮海经济区中心城市建设取得更大突破，保持经济持续健康发展和社会大局稳定，为高水平全面建成小康社会收官打下决定性基础，以优异成绩向新中国成立70周年献礼。

今年经济社会发展的主要预期目标是：地区生产总值增长6%左右，一般公共预算收入增长4.5%左右，固定资产投资增长6.5%左右，社会消费品零售总额增长8.5%左右，进出口总额和实际使用外资分别增长10%左右，全社会研发投入占比2.1%左右，居民收入增长高于GDP增长，居民消费价格涨幅控制在3%左右。实现这些目标任务，要重点抓好以下几方面工作：

（一）更大力度推动转型升级，构建现代产业新体系。坚持“工业立市、产业强市”不动摇，以创建国家老工业城市和资源型城市产业转型升级示范区为牵引，不断优化产业结构、强化项目建设、壮大实体经济，打造具有徐州特色的“6+6”先进制造业和“333”现代服务业体系。

做大做强新兴产业。以培育四大新兴主导产业为重点，排定年度工作要点、重点项目和企业培育库，力争新兴产业产值增长12%、占比提高2个百分点。装备与智能制造产业重点抓好徐工大型矿山机械、特种起重机械等项目建设，加快集聚整机和核心零部件项目，扶持徐工集团发展壮大，打造世界级工程机械研发制造基地。新能源产业加快实施薄膜太阳能电池、固体氧化物燃料电池等项目，提升光伏产业优势，力争在新能源汽车及关键零部件领域取得突破。集成电路与ICT产业大力推进半导体材料、装备、封装、测试及专用芯片制造项目集聚，发展第三代半导体材料、器件产业，打造自主可控、特色鲜明的集成电路产业高地。生物医药与大健康产业抓好重点领域化学新药和生物制药研发制造，做大医疗器械、医用材料、康体养生等优势产业。积极培育节能环保、新材料、安全科技等特色产业，前瞻布局5G通讯、数字经济等新业态，抓好大数据、云计算等新技术应用，促进“四新经济”蓬勃发展。加快创建全国质量强市示范城市。

改造提升传统产业。围绕提升层次、降低消耗、提高效益，引导企业大规模实施技术革新、产品更新、设备改造，深入推进四大行业布局优化和转型升级，让“老产业”焕发新生机。钢铁行业以高端化规模化为方向，加快产能整合和产品升级，推进重点钢铁企业扩容提质，大力发展精钢特钢。落实焦化行业转型升级方案，推进产能减量置换，推动产品精深加工、资源高效利用。整合水泥粉磨企业，促进水泥行业加速调整布局，加快向新型建材业转型。推进热电行业“上大压小”，有序实施清洁能源替代，淘汰30万千瓦及以上机组15公里半径范围内燃煤锅炉、落后燃煤小热电。

大力培育现代服务业。深入开展国家服务业综合改革试点，重点突破现代物流、金融服务、文化旅游产业，大力发展科技服务、电子商务、软件服务外包等新兴服务业，年内服务业投资增长8.5%以上。实施区域性多式联运中心建设三年行动计划，争取列入国家高铁物流重要节点城市，抓好徐州铁路物流园、苏宁电商智慧物流园等项目建设，规划建设城市东南片区物流市场集聚区，引导批发市场、商贸物流企业联合重组，力争物流行业营业收入增长10%以上。完善现代金融服务体系，深入实施招金引银战略，年内新签约金融服务机构50家，建设线上综合金融服务平台，扩大离岸金融市场服务，新设立创投企业10家、产业基金60亿元以上。编制《全域旅游发展规划》，提升云龙湖景区内涵，抓好方特欢乐世界、海洋极地世界等旅游项目建设，深化国家全域旅游示范区创建，促进旅游与文化、生态、农业等深度融合。

抓好招商引资和项目建设。围绕我市重点产业，绘制产业图谱和招商地图，做足延链、强链、补链文章，形成大项目顶天立地、中小项目铺天盖地的态势。国内聚焦北京、上海、浙江、粤港澳台，国外聚焦欧洲、美国、日韩等重点地区，策划举办更具针对性的推介活动，提高招商实效。建设专业化招商队伍，加强驻外招商人员配备，重奖招商有功人员。认真执行项目建设“六项制度”，持续推进“765”计划，建立项目信息化管理平台，完善观摩点评和推进考核机制，推动项目早开工、早竣工、早达产。加快实施当年投资1514亿元的205项重大产业项目，重点抓好天丝新材料、中铁磁悬浮、协鑫大硅片等当年实施项目，积极推进机器人制造谷、新型膜材料等前期项目。

发展壮大民营经济。全面落实支持民营经济健康发展32条措施，抓好减税降费等优惠政策落地，发挥政府采购引导作用，支持首台（套）等产品示范应用。建立各级领导包挂帮扶企业机制，真心实意为企业服务、和企业家交朋友，建立“亲”“清”政商关系。在行政服务大厅设立民营企业服务中心，及时解决企业反映的突出问题。制定发展壮大领军企业、高成长型企业、上市后备企业行动计划，抓好“四上”企业培育，对不同阶段企业提供精准服务。完善优秀企业家培树机制，弘扬企业家精神。继续抓好优秀企业家评选，营造全社会尊重企业家的浓厚氛围，让企业家更有地位、更有尊严、更有荣誉感！

（二)大力推进改革开放，释放经济发展新活力。站在改革开放40周年新起点，以改革再深化激活发展机制，以开放再扩大拓展发展空间，为高质量发展注入强劲内生动力。

全面深化重点领域改革。贯彻落实“巩固、增强、提升、畅通”八字方针，深入推进供给侧结构性改革。完成市县机构、生产经营类事业单位和承担行政职能的事业单位改革。抓好“不见面”审批标准化建设，深化“3550”改革。推开“证照分离”改革，实行“照后减证”“证照联办”。实施新一轮市属国企改革，推动国资公司市场化转型，稳妥推进徐工混改。深化农村集体产权制度、承包土地经营权抵押贷款、社区股份合作制等改革，持续激发农村发展活力。抓好市区农商行改革重组，切实化解互联网金融、P2P网贷等领域风险，严厉打击非法集资，稳妥化解政府债务，坚决守住不发生系统性金融风险的底线。抓好第四次全国经济普查、第三次国土调查。

推动全方位对外开放。立足打造“一带一路”高水平产业集聚区、经贸合作高地和江苏省向西开放门户，进一步扩大开放，打造“东西南北”开放大通道，形成立体开放体系。抓好徐州综保区、新沂保税物流中心运营，提升电子口岸便利化水平，保障中欧班列稳定运营，加快国际邮件互换局、“外贸港”、“无水港”建设。启动观音机场T1航站楼改造，增加国际客运航线，力争旅客吞吐量突破300万人次。主动应对国际贸易摩擦，鼓励机械制造、光伏、采矿业等优势产业“走出去”，推进工程机械及关键零部件、机床设备等进口交易中心建设，支持徐工巴西工业园申报省级境外产业合作集聚区。推动徐州经济技术开发区进位争先、高新区提档升级，支持邳州、沛县、新沂开发区争创国家级开发区。培育跨境电商、外贸综合服务平台等贸易新业态新模式，争创国家级服务外包示范城市、国家跨境电商综合试验区。加强国际友城合作，扩大人文交流，提升徐州国际知名度。

深入抓好营商环境建设。聚焦“五最”目标，扎实开展“营商环境提升年”活动。对照世界银行和国家营商环境评价标准，构建我市营商环境建设指标体系，积极开展第三方评估，力争各项评价指标明显提升，争取进入2019年部分地级市营商环境试点评价范围。全面梳理社会各界反映强烈的营商环境问题，逐项整改落实。开展“万家企业评机关”活动，查处曝光一批反面典型，让企业和群众切实感受到政府服务在优化、效率在提升。

（三）持续强化创新驱动发展，打造加快转型新动能。科技是第一生产力，人才是第一资源，创新是第一动力。深入实施创新驱动发展战略，更高层次、更大范围集聚创新资源要素，着力建设国家创新型城市。

打造一流创新载体。加快建设“一城一谷一区一院”四大创新平台，理顺科学管理和高效运行机制，打造科技创新“引领极”。大力推进科技企业孵化器、加速器、众创空间等平台专业化孵化、市场化运作，聚焦新兴产业领域、国外先进科技成果实施精准孵化、离岸孵化，打造科技企业孵化育成全链条。进一步完善产学研协同创新机制，组织好大院大所对接活动，招引新型研发机构落户，承接转化产业技术创新项目。建成国家工程机械、省特种机器人质检中心。

集聚更多创新人才。紧扣产业需求绘制“人才地图”，办好中国徐州·上海人才峰会、“赢在徐州”创新创业大赛等招才引智品牌活动，大力招引领军人才和团队。建立健全领导干部联系产业人才、包挂重点企业制度，深入开展“彭城英才蓝卡行动”，对高层次人才提供全方位服务。开展“百企名校行”活动，抓好大学生创业园、人力资源服务产业园和人才公寓建设，年内吸引2.5万名高校毕业生在徐州发展。注重高技能人才和高素质产业工人培养，推行企业新型学徒制，拓宽就业技能、岗位技能培训补贴范围，年内开展订单式培训1万人以上。

构建优质创新生态。全面落实深化科技体制改革各项举措，建立市县联动的政策落实跟踪机制。大力培育高新技术、科技小巨人、瞪羚和独角兽企业，支持规模以上企业与高校院所共建“一站两院三中心”，年内新增高新技术企业210家以上。提升技术产权交易中心功能，支持海内外高校院所在徐设立技术转移中心，吸引国内外科技成果在徐州转化。完善科技金融服务体系，抓好科技金融担保，引导更多风投基金、天使基金投入科技成果产业化。深化国家知识产权示范市建设，实施高质量专利培育计划，提升科研产出质量和市场应用水平。

（四）不断增强集聚辐射能力，实现中心城市建设新突破。聚焦做强做美做优现代化中心城市，实施六大类100项城建重点工程，努力提升城市首位度，带动城乡融合发展、区域协同发展。

完善城市功能设施。编制城市总体规划（2020-2035），完善城市东南片区、西北片区、会展中心片区、圣戈班片区规划和大郭庄片区城市设计，积极争取全国国土空间规划试点。轨道交通1号线、迎宾大道高架开通使用，加快轨道交通2号、3号线建设，抓好城东大道高架和三环南路、徐丰路、徐韩公路快速化改造等道路建设，建成徐宿淮盐高铁徐州段，加快徐连高铁、疏港铁路等项目建设，打造内畅外联快捷交通网络。突出构建大市场、大物流体系，加快培育淮海经济区货物集散中心，大力发展枢纽经济。高标准建设淮海会展中心、国际会议中心、市民文化活动中心、公共卫生中心等功能性项目，进一步优化公共服务资源布局。抓好淮海经济区金融服务中心建设，大力招引数据中心、清算中心等服务机构，提升金融后台服务功能。实施主城区老旧小区二次供水改造、建筑屋顶清理改造等工程。完善新城区教育、医疗、公共交通、便民服务等功能配套，加快发展城市型经济，实现新城区产城融合发展。

提升城市品质内涵。深入挖掘两汉文化、运河文化、红色文化、乡村文化、彭祖文化等特色文化内涵，抓好大运河文化带徐州段建设，实施文庙、戏马台等历史文化街区修复工程，打造更多带有徐州记忆的“文化地标”，放大“楚韵汉风”的独特魅力。持续推进城市双修，抓好九里山、托龙山等山体修复，启动1680万㎡棚户区改造、完成58个拆迁扫尾项目。加快建设给排水、综合管廊等市政基础设施，新增停车泊位1万个以上。实施卧牛山、桃花源湿地等公园建设，积极申办第十三届中国国际园林博览会，扩大“徐派园林”影响力。统筹推进城市出入口、建筑外立面、背街小巷整治，推动城市管理精细化、规范化。扎实抓好智慧城市建设，重点在智慧交通、智慧医疗、智慧城管等领域求突破。

健全新型城镇体系。加快推进以人为核心的城镇化，持续完善新型城镇体系。支持5座县城围绕中等城市定位，完善功能配套，加快产城融合。抓好9个中心镇试点创建、15个乡村振兴先导镇创建和特色小镇培育，推进其他镇加快改善镇容镇貌、补齐基础设施短板，年内所有镇达到“六无四净”标准、城镇污水处理率达到92.3%。创建15个新型社区，抓好基本公共服务标准化建设。

促进区域融合发展。落实淮海经济区协同发展机制，推进徐淮、徐宿快速通道等39个互联互通项目，构建以徐州为中心的“1小时交通圈”。加强产业规划对接，深化产业分工协作，打造跨省际现代产业合作新体系。协同开展跨省大气污染防治，推进生态环境保护联防联控。创新教育、医疗卫生、文化、体育、旅游等城际合作机制，促进公共服务共建共享。

（五）深入实施乡村振兴战略，开创“三农”工作新局面。按照“产业兴旺、生态宜居、乡风文明、治理有效、生活富裕”总要求，加快推动农业全面升级、农村全面进步、农民全面发展，走出一条具有徐州特色的乡村振兴之路。

大力发展现代农业。坚持质量兴农、绿色兴农、品牌强农，抓好国家农业可持续发展试验示范区暨农业绿色发展先行区建设，支持发展多种形式的适度规模经营，建设19个农业产业集群。年内创建省级以上农产品加工集中区2家，培育市级以上农业龙头企业20家，提升粮食加工转化率。加强农业品牌建设，打造“1+4+N”农业品牌体系。整合组建市农业科技推广服务中心，完善农业科技首席专家顾问制度，形成覆盖全市的农业科技推广服务体系。继续抓好上海蔬菜外延基地建设，搭建更多的农产品产销对接平台。加快发展农村电子商务，培育更多农村电商品牌。稳定粮食产量，抓好优良品种推广，建设高标准农田45万亩，新建改造设施农业10万亩，农业机械化水平提高到85%以上。大力培育新型职业农民，促进资本下乡、能人返乡、企业兴乡，打造乡村振兴的生力军。

坚决打赢脱贫攻坚战。紧盯“两不愁三保障”目标，综合推进产业、就业创业、搬迁、医疗健康、综合保障扶贫，坚持“一户一措施、一户一项目”，年内实现7.1万建档立卡低收入人口脱贫。以搬迁扶贫为重点，抓好新型农民集中居住区建设，有序推进村庄搬迁，不断改善农村居住环境。年内完成5886户低收入农户危房改造。支持经济薄弱村发展，拓宽村集体增收渠道，推动51个经济薄弱村集体经营性收入超过18万元。大力推进农田水利、交通网络、土地整治等“八项工程”，强化湖西片区、黄墩湖滞洪区、黄河故道沿线扶贫攻坚，实现丰县、睢宁县省级贫困县“摘帽”。坚持扶贫与扶志扶智相结合，用好阳光扶贫监管平台，优化扶贫资源配置，构建精准扶贫长效机制。

着力改善乡村面貌。深入实施农村人居环境整治，扎实推进公共空间治理。抓好农村生活垃圾治理，提升垃圾保洁作业体系覆盖率和集中收运、无害化处理率。加快郑集河输水工程建设，加大河道、河塘疏浚力度，推进镇级污水处理厂达标运行，年内行政村生活污水治理覆盖率达到40%以上。扎实开展“厕所革命”，抓好行政村冲水式公厕建设，新建户厕6.68万座、修缮11.3万座。深入治理面源污染，年内秸秆综合利用率达到95%、畜禽粪污综合利用率达到75%。高度重视饮水安全，基本实现城乡供水一体化入户全覆盖。新建改建农村道路500公里、桥梁100座，实现行政村双车道四级公路全覆盖，推进村内道路“户户通”。抓好特色田园乡村、美丽宜居乡村建设，建成35个省市级特色田园乡村、200个省级美丽宜居乡村。推广“马庄经验”，抓好农村精神文明建设，提高农民素养，丰富农民文化生活。

（六）切实抓好生态文明建设，彰显生态宜居新形象。牢固树立“绿水青山就是金山银山”的理念，坚决打好污染防治攻坚战，全力抓好中央环保督察“回头看”反馈问题整改，实现环保指标和群众实际感受“双提升”。

打赢蓝天碧水净土保卫战。强力推进大气污染防治，大力调整优化产业、能源、运输结构，深化“六项治理”，严控煤炭消费总量，提高铁路、水运运输比例，淘汰国Ⅱ汽油车5000辆、国Ⅲ柴油车5000辆，加强道路、工地、堆场、码头扬尘管控，全力削减挥发性有机物，确保PM2.5平均浓度控制在58μg/m³以内、优良天数比率达到62%以上。抓好市区雨污管网建设、奎河水环境综合整治、黑臭河道景观提升和水系贯通工程，基本消除城市规划区黑臭水体；实施生态河湖行动计划，抓好河湖圈圩集中整治，全面消除劣Ⅴ类水体。加强土壤污染治理修复及风险管控，加快循环经济产业园建设，抓好第二垃圾焚烧厂、雁群垃圾填埋场等处理设施建设，提升固体废物集中处置能力。

加快推进生态保护修复。实施山水林田湖草系统保护与修复，构建“两轴两湖多片区多点”生态空间结构。推广潘安湖修复治理模式，启动城北等6万亩采煤沉陷地治理，支持沛县创建国家重点采煤沉陷区综合治理试点。编制矿山环境恢复与治理规划，抓好绿色矿山建设。实施绿化造林5万亩，科学调整林木结构，保证林木覆盖率稳定在30.1%、建成区绿化覆盖率稳定在43.8%以上。推进采煤沉陷区、采石宕口生态修复“徐州标准”国际化。

健全生态文明体制机制。颁布实施《徐州市大气污染防治条例》。制定生态环境准入清单，严格限制高能耗高污染项目落地。坚持上门服务、精准治污、差别化管理的理念，创新环境执法监管机制，完善环境网格化监管体系，健全污染防治综合监管平台，实现环境监测和执法监管有效衔接。加强环保领域信用体系建设，探索实行企业环保承诺制，建立生态环境损害赔偿和高风险领域环境污染强制责任保险制度，强化企业主体责任和环保责任追究。

倡导绿色低碳生活方式。大力培育生态文化，壮大环保社会组织和志愿者队伍，广泛引导公众参与。推行绿色出行、绿色消费、绿色餐饮，抓好绿色学校、绿色社区建设，建成区新增公交全部使用新能源或清洁能源车辆，引导全社会形成绿色低碳、文明健康的生活方式。开展生态文明建设示范创建，加快创建一批国家级和省级生态文明示范县、镇、村。

（七）千方百计增进民生福祉，顺应群众美好生活新期盼。保障和改善民生只有起点、没有终点。我们将坚持富民优先、民生共享，进一步完善社会建设“十二大体系”，让全市人民生活得更加安心、顺心、舒心。

积极拓宽群众增收渠道。以发展产业、扩大就业、促进创业为抓手，深入实施八项富民工程，细化落实“富民33条”，促进群众收入持续增长。制定新一轮促进就业扶持政策，开展“就业援助月”“民营企业招聘周”等专项活动，扩大就业困难人员援助范围，年内新增城镇就业9万人、农村劳动力转移4万人。大规模开展创业培训和职业技能培训，实施大学生创业、“彭城妇创”等创业帮扶活动，促进高校毕业生、失业下岗人员、农民工和退伍军人等重点群体多渠道就业创业。

加快完善社会保障体系。深入实施全民参保计划，提高社保覆盖面和待遇水平，将重度失智人员纳入基本照护险保障范围。推进城乡居民医保政策一体化，扩大与周边地区门诊直接结算范围。落实城乡居民低保、农村五保、城市“三无老人”和孤儿供养标准增长机制，实施困境青少年关爱工程，为3.94万病残儿童提供救助。抓好“残疾人之家”建设，完成4380户贫困残疾人家庭无障碍改造。抓好公租房分配入住及盘活转用，落实好城市低保特困家庭、低收入困难家庭等重点群体住房租赁补贴，推动住房公积金归集扩面。

不断丰富公共服务供给。坚持教育优先发展，加快实施学校建设，年内建成幼儿园53所、中小学校40所；强化教师队伍建设，提高师资保障水平，年内补充中小学教师7000人；加强教育内涵建设，义务教育学校标准化比例达到90%、公办高中优质比例达到80%；推动徐州幼专新校区建设和专升本、徐州工程学院建设徐州大学，支持驻徐高校创建“双一流”大学；大力发展职业教育，促进产教融合。聚焦打造区域医疗高地，市第一人民医院新院、北区股份制医院年内建成投入使用，抓好市儿童医院、康复医院等专科医院新院建设；大力引进高层次医疗专家团队和专科人才，支持眼科、麻醉、新生儿等重点专科建设；推动新一轮基层基本公共医疗卫生体系提档升级，建设262个省级示范标准村卫生室。深化全国居家和社区养老服务改革试点，新改扩建100个社区居家养老服务中心，推动农村敬老院转型升级，办好老年大学。高水平举办国际马拉松赛、国际武术大赛等体育赛事，促进全民健身、竞技体育和体育产业融合发展。抓好文化惠民服务项目，加快基层公共文化设施建设，办好第二届淮海书展、第六届文博会，打造“书香徐州”，让市民生活更加丰富多彩。

全面加强和创新社会治理。巩固全国文明城市创建成果，抓好新时代文明实践中心建设，开展“学雷锋榜样、做徐州好人”主题实践活动，发扬淮海战役精神，抓好普法和诚信教育。深入开展扫黑除恶专项斗争，深化平安法治徐州建设。推进社会治理“一张网”二期工程，年内县镇村网格化服务管理中心规范化建成率分别达到100%、90%和70%。实施“雪亮工程”，初步建成公共安全监控联网应用体系。深入开展“百千万”大走访，加快信访积案化解。提高基层治理能力，加强社区服务中心建设，推广全科社工服务模式。落实安全生产责任制，及时消除各类事故隐患，突出抓好道路交通安全，健全“以防为主”的应急管理体系，提高各类事故处置能力。做好双拥共建和优抚安置，实现双拥模范城“九连冠”。

各位代表，办好民生实事是政府最大的政绩。今年我们在广泛征集民意的基础上，围绕群众最关心的教育提质、健康惠民、安居宜居、公共安全等11个方面，安排了46件实事。我们将把这些实事办成办好，向人民群众交上满意的答卷！

三、建设人民满意政府

新形势、新目标、新任务对加强和改进政府工作提出了更高要求。我们一定不忘初心、牢记使命，以时不我待的紧迫感、舍我其谁的责任感，大力提升推动发展能力、改革创新能力、攻坚克难能力，加快政府职能转变，努力建设人民满意政府。

（一）加强政治建设，提高政治站位。旗帜鲜明讲政治，增强“四个意识”，坚定“四个自信”，坚决维护习近平总书记的核心地位，坚决维护以习近平同志为核心的党中央权威和集中统一领导，始终同以习近平同志为核心的党中央保持高度一致。深入学习贯彻习近平新时代中国特色社会主义思想，自觉用以武装头脑、指导实践、推动工作。加强学习和调查研究，不断解放思想，创造性贯彻落实党中央国务院、省委省政府和市委各项决策部署，确保政令畅通、令行禁止。

（二）全面履职尽责，提升效率效能。坚持把狠抓落实作为政府工作的核心，完善月度工作例会、重点工作调度、重点难点事项协调等机制，强化绩效管理、政务督查和行政问责，以“钉钉子”精神将重点工作抓紧盯牢、落细落实。强化政府工作人员的公仆意识、责任意识、效率意识，坚决整治庸懒散拖、落实不力、敷衍塞责等行为。用好用活“三项机制”，鼓励基层先行先试，创造更多的徐州经验、徐州典型。深入推进行政审批服务、公共资源交易等平台建设，提升“互联网+政务服务”水平，推动基层政务服务标准化规范化，打造优质高效的政务环境。

（三）坚持依法行政，建设法治政府。强化法治思维，严格依法办事，把依法行政贯穿于政府工作全过程。健全依法决策机制，发挥新型智库作用，加强与人大代表、政协委员的沟通联络，广泛听取各民主党派、工商联、无党派人士和人民团体意见，提高决策科学化民主化法治化水平。坚持严格规范公正文明执法，推进综合执法，推动行政执法和刑事司法有效衔接。自觉接受市人大及其常委会法律监督和工作监督、政协民主监督，重视司法、舆论、社会监督。深化政务公开，加强信息发布，及时回应社会关切，让行政权力在阳光下运行。

（四）持续改进作风，抓好反腐倡廉。认真贯彻执行中央八项规定精神及省、市委贯彻落实具体办法，深入落实党风廉政建设“一岗双责”，紧盯不敬畏、不在乎、喊口号、装样子的问题，坚决破除形式主义、官僚主义。坚持过紧日子思想，厉行勤俭节约，从严管理“三公经费”，把有限的资源和财力用在推动发展、改善民生上。突出公共权力运行、公共资源配置、公共资金分配等重点领域，严肃查处各类腐败问题，切实解决损害群众利益的不正之风。深化标本兼治，保持惩治腐败高压态势，着力打造干部清正、政府清廉、政治清明的廉洁政治“三清示范区”。

各位代表，潮起海天阔，扬帆正当时。让我们紧密地团结在以习近平同志为核心的党中央周围，在市委的坚强领导下，团结拼搏、负重奋进，在高水平全面建成小康社会、打造淮海经济区中心城市、建设“强富美高”新徐州的征程上阔步前进，以优异成绩向新中国成立70周年献礼！
